# Impact of Blood Volume on Pathogen Detection and Time to Positivity in Paediatric Blood Cultures Using BACT/ALERT 3D Automated Systems

**DOI:** 10.7759/cureus.88454

**Published:** 2025-07-21

**Authors:** Nikhil K Yadav, Nikhil Raj, Vikramjeet Singh, Mohd Saquib, Krishna Yadav, Dipti Agarwal, Anupam Das, Manodeep Sen, Jyotsna Agarwal

**Affiliations:** 1 Microbiology, Dr. Ram Manohar Lohia Institute of Medical Sciences, Lucknow, IND; 2 Medical Microbiology, Sanjay Gandhi Postgraduate Institute of Medical Sciences, Lucknow, IND; 3 Pediatrics, Dr. Ram Manohar Lohia Institute of Medical Sciences, Lucknow, IND

**Keywords:** bact/alert 3d system, bloodstream infection (bsi), blood volume, paediatric blood culture, time to positivity (ttp)

## Abstract

Background

Bloodstream infections (BSIs) are a major cause of morbidity and mortality in paediatric populations. Early and accurate pathogen detection is crucial for prompt therapy. Blood volume is a key factor influencing culture sensitivity and time to positivity (TTP). This study evaluated the impact of blood volume on microbial recovery and TTP in paediatric blood cultures using the BACT/ALERT 3D automated system (bioMérieux, Marcy-l'Étoile, France).

Methods

In this prospective study (April 2023-September 2024), 160 paediatric blood culture bottles were analysed. Blood volume was calculated using pre- and post-inoculation weights. Bottles were classified as optimal (>0.5 mL) or suboptimal (<0.5 mL). TTP and pathogen recovery were compared.

Results

Among 160 paediatric blood cultures, 78 (48.75%) flagged positive. Coagulase-negative *Staphylococci* (CoNS) were the most common isolates, followed by *Candida *spp. and *Klebsiella pneumoniae*. Median TTP was 18.13 h. No significant difference in pathogen recovery between optimal and suboptimal volume groups was observed. Gram-negative isolates had the shortest TTP (median: 11.39 h).

Conclusion

While adequate blood volume remains important, suboptimal volumes may still yield clinically significant pathogens with acceptable detection times, supporting the feasibility of low-volume cultures in neonates. TTP may serve as an adjunct marker for early identification of pathogenic organisms.

## Introduction

Globally, bloodstream infections (BSIs) constitute a major cause of mortality [1]. Mortality rates can be significantly decreased, and antibiotic misuse can be avoided with early diagnosis and prompt beginning of suitable antimicrobial treatment [2]. Therefore, accurate and timely interpretation of blood culture results is essential for initiating appropriate treatment and improving patient outcomes [3].

Bacteraemia detection depends on factors like bacterial load, blood volume, transport time, culture sets, and skin disinfection, with blood volume being the most critical for optimal microbial recovery [4,5]. Studies have shown that blood cultures often fail to detect pathogens in paediatric patients with sepsis. In two well-documented studies, only 25% to 26% of children admitted to intensive care units with clinical signs of sepsis had positive blood cultures [6,7]. The blood volumes typically collected for culture in both infants and older children are often insufficient for accurate and rapid pathogen detection, particularly when bacteria are present in low concentrations [8].

Paediatric blood cultures have traditionally been performed with as little as 1 mL of blood [8]. However, recent studies indicate that culturing just 0.2 mL of blood achieves 95% sensitivity compared to 2 mL in infants up to 12 months old [9].

Advancements in blood culture systems are challenging long-held beliefs about BSI detection, with studies suggesting that a full five-day incubation period may not be necessary for identifying clinically relevant pathogens [10,11]. Blood volume is crucial for optimizing microorganism detection, but the use of paediatric versus standard blood culture media remains debated. Time to positivity (TTP) reflects bacterial load and is a key parameter in determining the presence of infection [12]. Clinicians often use TTP as a prognostic marker to assess disease severity and guide treatment decisions.

The objective of this study is to evaluate the volume of blood drawn for automated blood culture testing and its impact on microbial isolation rates and TTP. The success of blood culture depends on multiple factors, including blood volume, dilution ratio, timing of sample collection, bacteraemia level, number of cultures obtained, and sampling techniques [13]. Studies indicate that up to 60% of positive paediatric blood cultures involve low bacterial concentrations (≤10 CFU/mL) [8]. One of the main challenges in neonatal intensive care units (NICUs) is obtaining an adequate blood sample volume, particularly in preterm neonates. Difficulty in venous access and the urgency of initiating antibiotic therapy often lead to suboptimal blood culture volumes.

## Materials and methods

This was a prospective observational study conducted over 18 months (April 2023- Sep 2024) to evaluate the impact of blood volume on the detection of BSIs using the automated blood culture system (BACT/ALERT 3D [bioMérieux, Marcy-l'Étoile, France]). A total of 160 blood culture samples from the Department of Paediatrics were analyzed. All received samples were included except those contaminated with aerobic spore bearers or diphtheroids.

Determination of blood culture volume 

When the blood culture bottles arrived at the microbiology lab, they were measured using a digital weigh balance (readability: 0.01 g to 1000 g). Blood volume was calculated using standard equations (Table 1) while accounting for the absence of the original cap and barcode sticker and the presence of an additional patient barcode. Blood density (1.06 g/mL) was taken into consideration when converting the measured blood weight (g) to volume (mL). The appropriateness of blood culture bottles was assessed based on blood volume (Table 1). 

**Table 1:** Formula for measuring blood volume and classifying blood culture bottles

Parameter	Equation
Weight before filling (g)	Unfilled bottle weight + bottle cap weight + patient barcode weight
Weight after filling (g)	Filled bottle weight + patient barcode sticker weight + bottle cap weight
Blood drawn (g)	Weight after filling - weight before filling - patient barcode sticker - cap weight
Blood volume (mL)	Blood drawn (g) × blood density (1.06 g/mL)
Optimal filled bottles	>0.5 mL
Suboptimal filled bottles	<0.5 mL

Inoculation of automated blood culture and antimicrobial susceptibility testing

Blood culture bottles were scanned in the BACT/ALERT 3D blood culture system and placed in the designated chamber for incubation. After incubation, when a blood culture bottle was flagged positive, the TTP was recorded. Following a 24-hour incubation period at 37°C, the bottle was plated on Blood Agar and MacConkey Agar. Following incubation, the culture plates were examined to confirm microbial growth, which was further identified using biochemical tests and VITEK-MS (bioMérieux, Marcy-l'Étoile, France). Subsequent processing was carried out as per standard microbiological protocols. Bacterial susceptibility testing was performed using the Kirby-Bauer disc diffusion and VITEK 2 COMPACT (bioMérieux, Marcy-l'Étoile, France) testing in accordance with the Clinical and Laboratory Standards Institute (CLSI) M100 (2023) guidelines. 

Statistical analysis

Descriptive statistics were used to summarize demographic and clinical characteristics, with categorical variables expressed as frequencies and percentages, and continuous variables reported as median (interquartile range, IQR) or mean ± standard deviation (SD), as appropriate. The median TTP was calculated for each microbial group. T-tests were applied to compare the mean TTP values among different categories of isolates (Gram-positive, Gram-negative, and *Candida* spp.). A p-value of <0.05 was considered statistically significant. The chi-square (χ²) test was employed to assess differences in pathogen isolation rates between optimally and suboptimally filled blood culture bottles. 

## Results

A total of 160 paediatric patients (age range: 1 day-12 years, median: 2 years) were included. The study population included a wide age spectrum of the paediatric population, from newborns to pre-adolescents. Based on age, 18 (11.3%) were neonates (0-28 days), 20 (12.5%) were infants (>28 days to 1 year), 46 (28.8%) were toddlers (1-3 years), 28 (17.5%) were preschool-aged children (>3 to 5 years), and 48 (30.0%) were in the school-age group (>5 to 12 years) as shown in Table 2. Of these 160 patients, 97 (60.24%) were from rural areas and 63 (39.75%) from urban settings. Of the total 160 patients, 94 (59%) were male patients and 66 (41%) were female patients. Of the 160 children, 3 (1.9%) had a birth weight of less than 1.5 kg, 16 (10.0%) had a birth weight between 1.5 and 2.5 kg, and 141 (88.1%) had a birth weight of more than 2.5 kg. Common symptoms included fever, jaundice, respiratory distress, cough, and abdominal pain. 

**Table 2:** Demographic characteristics of study participants

Characteristic	Category	n (%)
Sex	Male	94 (59%)
	Female	66 (41%)
Age group	Neonates (0-28 days)	18 (11.3%)
	Infants (>28 days–1 year)	20 (12.5%)
	Toddlers (1-3 years)	46 (28.8%)
	Preschoolers (>3-5 years)	28 (17.5%)
	School-aged (>5-12 years)	48 (30.0%)
Birth weight	<1.5 kg	3 (1.9%)
	1.5-2.5 kg	16 (10.0%)
	>2.5 kg	141 (88.1%)

Among 160 blood culture bottles analysed, 82 (51.25%) were sterile, while 78 (48.75%) were flagged as positive. The median TTP for flagged blood cultures was 18.13 h (range: 4.21-107.2 h). By 24 h, 59 (75%) blood cultures flagged positive; similarly, by 36 h, 66 (85%) blood cultures, and by 48 h, 73 (94%) blood cultures, respectively, were flagged positive. Gram-negative bacteria had the shortest TTP (median: 11.39 h), while *Micrococcus *spp. had the longest (39.25 h) (Table 3). All coagulase-negative* Staphylococci *(CoNS) isolates were associated with clinical signs of sepsis and had shorter TTP compared to contaminants, confirming their pathogenic role.

**Table 3:** TTP of bacterial and fungal isolates in blood culture TTP: time to positivity; IQR: interquartile range; SD: standard deviation; h: hours. Note: TTP is expressed as median (with IQR), and mean ± SD. p-Values were calculated using the t-test to compare the mean TTP between organism groups. A p-value <0.05 was considered statistically significant.

Serial number	Organism	Median TTP (h)	IQR (h)	Mean ± SD (h)	t-Value	p-Value
1	Gram-positive isolates	18.17	14.38-21.49	19.4 ± 7.23	5.4336	0.0001
2	Gram-negative isolates	11.39	5.59-16.64	13.79 ± 10.73	3.4539	0.0023
3	*Candida *spp.	18.45	14.11-23.30	27.26 ± 24.97	1.5650	0.1319
4	Micrococcus	39.25	19.36-67.41	44.10 ± 27.3	-	-

Out of the 160 bottles, 17 (10.62%) bottles had suboptimal blood volume (less than 0.5 mL) and 143 (89.37%) bottles had an optimal volume (above 0.5 mL). The average TTP and pathogen isolation rate from blood culture bottles with and without an appropriate blood volume filling are shown in Table 3. The difference in the isolation rate between optimally filled and suboptimally filled blood culture bottles was statistically insignificant (p = 0.8031) (Table 4).

**Table 4:** Average TTP and pathogen isolation rate from blood culture bottles with and without an appropriate blood volume filling TTP: time to positivity. Note: The chi-square test was used to compare the proportions of true pathogen isolation between groups. The calculated chi-square (χ²) values and the corresponding p-values are also shown. A p-value <0.05 was considered statistically significant.

Blood volume	No of blood culture bottles flagged positive (N)	Average TTP (h) for pathogens	No of true pathogens (n)	Isolation rate (n/N) %	Chi-square (χ²) value	p-Value
Suboptimum (0.5 mL)	12	21.60	11	91.66	0.062	0.8031
Optimum (0.5-1 mL)	66	24.24	54	81.81		

Among 160 blood culture bottles, 78 (48.75%) flagged positive, while 82 (51.25%) remained sterile. Of these 78 positive cultures, 43 (55.12%) contained Gram-positive bacteria, 11 (14.10%) had Gram-negative bacteria, 11 (14.10%) grew *Candida, *and 13 (16.6%) were contaminants (*Micrococcus *spp.). 

**Figure 1:**
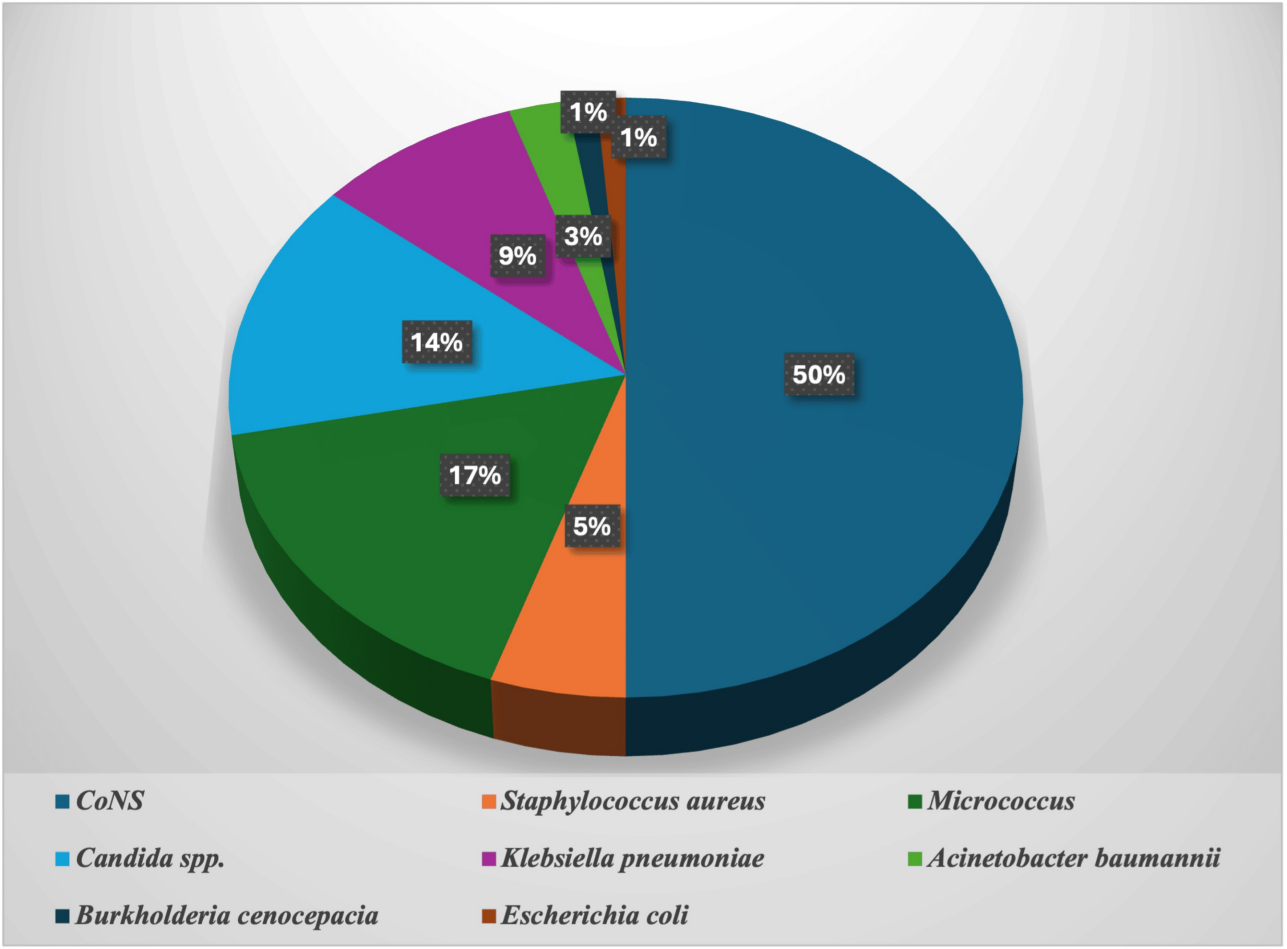
Distribution of microorganisms isolated from positive blood culture bottles CoNS: coagulase-negative *Staphylococci.*

Of the 78 positive blood culture bottles, 43 (55.12%) showed growth of Gram-positive bacteria, 11 (14.10%) had Gram-negative bacteria, 11 (14.10%) grew fungi, and 13 (16.6%) bottles showed growth of contaminants (*Micrococcus* spp.). Distribution of bacterial and fungal isolates was as follows (Figure 1): CoNS were the most frequently isolated organisms, accounting for 39 (50%) cases followed by *Candida* spp., in 11 (14.1%) cases, *Klebsiella pneumoniae* was isolated in 7 (9%) cases, *Staphylococcus aureus* in 4 (5.1%) cases, and *Acinetobacter baumannii* in 2 (2.6%) cases. Additionally, *Escherichia coli* and *Burkholderia cenocepacia* were each identified in 1 (1.3%) case.

**Figure 2:**
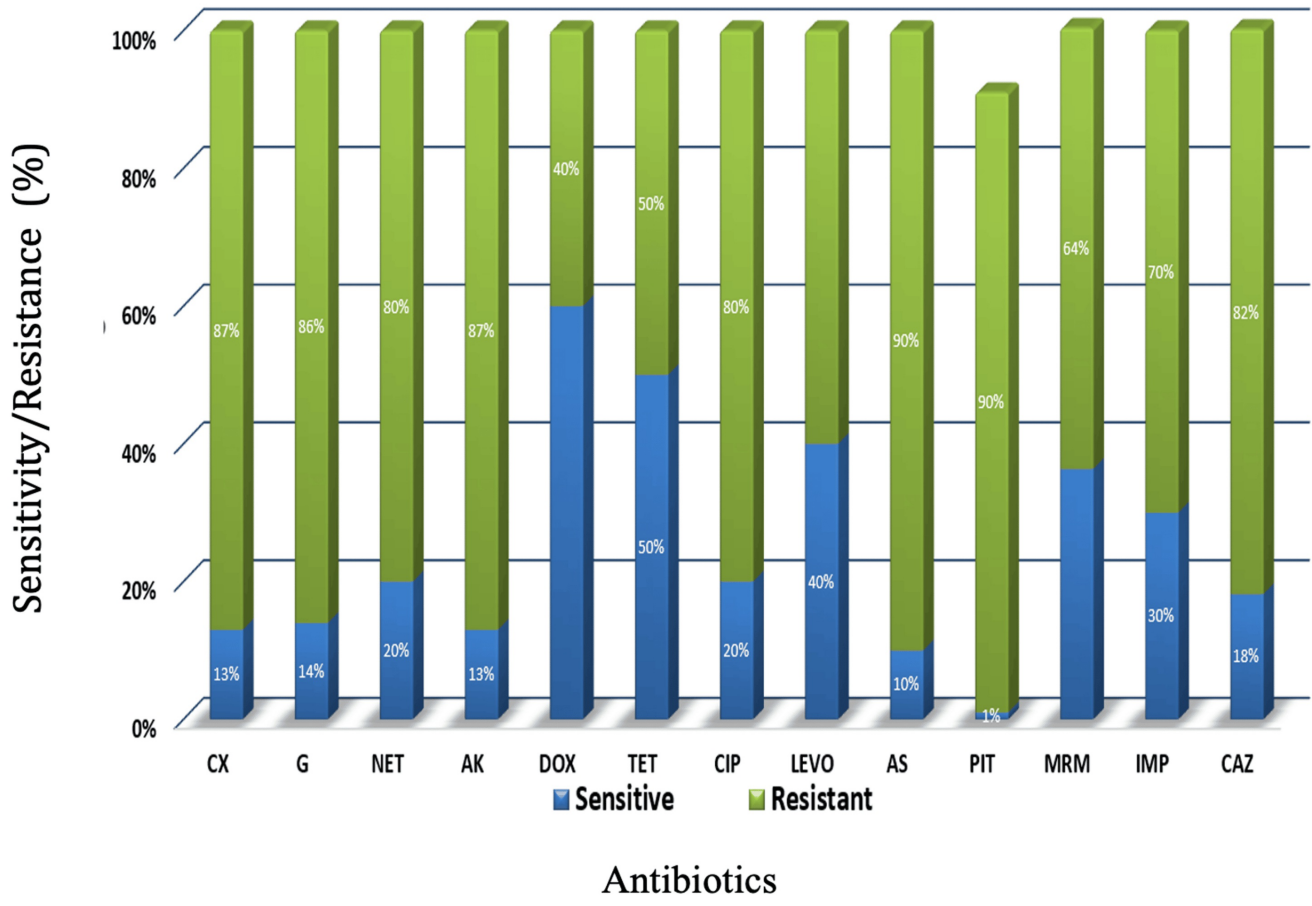
Antimicrobial agents tested for Gram-negative bacteria isolates and their resistance pattern PIT: piperacillin-tazobactam; A/S: ampicillin-sulbactam; AK: amikacin; CX: cefoxitin; G: gentamicin; CAZ: ceftazidime; NET: netilmicin; CIP: ciprofloxacin; IPM: imipenem; MRM: meropenem; LEVO: levofloxacin.

Among 11 isolates of Gram-negative bacteria (Figure 2), resistance was observed as follows: piperacillin-tazobactam in 11 (100%) isolates, ampicillin-sulbactam in 11 (100%) isolates, amikacin in 10 (90%) isolates, cefoxitin in 9 (87%) isolates, gentamicin in 9 (86%) isolates, ceftazidime in 9 (82%) isolates, netilmicin in 9 (80%) isolates, ciprofloxacin in 9 (80%) isolates, imipenem in 8 (70%) isolates, meropenem in 7 (64%) isolates, and levofloxacin in 7 (60%) isolates.

**Figure 3:**
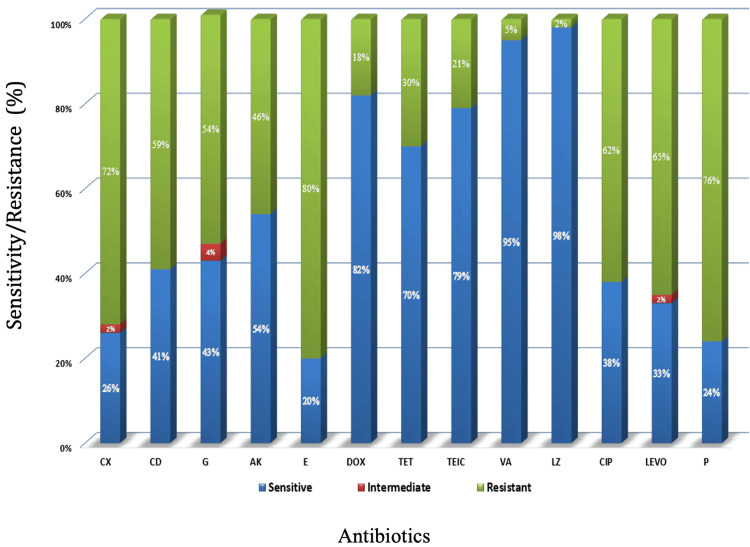
Antimicrobial agents tested for Gram-positive bacteria isolates and their resistance pattern P: penicillin; CX: cefoxitin; LE: levofloxacin; CIP: ciprofloxacin; CD: clindamycin; G: gentamicin; AK: amikacin; TET: tetracycline; TEIC: teicoplanin; VA: vancomycin; LZ: linezolid.

Among 43 isolates of Gram-positive cocci (Figure 3), resistance was observed as follows: erythromycin in 34 (80%) isolates, penicillin in 33 (76%) isolates, cefoxitin in 31 (72%) isolates, levofloxacin in 28 (65%) isolates, ciprofloxacin in 27 (62%) isolates, clindamycin in 25 (59%) isolates, gentamicin in 23 (54%) isolates, amikacin in 20 (46%) isolates, tetracycline in 13 (30%) isolates, teicoplanin in 9 (21%) isolates, vancomycin in 2 (5%) isolates, and linezolid in 1 (2%) isolate.

## Discussion

Blood volume is a critical factor influencing the recovery of microorganisms in blood cultures. Given that microorganisms are often present in very low concentrations, ranging from fewer than 1 to 10 cfu/mL, submitting an insufficient blood volume increases the risk of missing them, potentially leading to false-negative results [14,15]. The most reliable method for identifying bacteraemia in unwell preterm babies and neonates is still blood culture [16]. It makes testing for antibiotic susceptibility and pathogen identification easier, enabling accurate diagnosis and appropriate adjustments to empiric antibiotic therapy. Conversely, a negative result allows for the timely discontinuation of antibiotics, reducing the risk of selecting for resistant bacteria.

Studies on the optimal blood volume for paediatric blood cultures have produced conflicting results. While some suggest that inadequate sample volume and an insufficient number of cultures may lead to false-negative results, others argue otherwise. Consequently, the ideal sample volume for neonatal blood cultures remains a subject of debate. Findings from the present study indicate that using a single blood culture bottle with more than 0.5 mL of blood yields a high rate of pathogen isolation. Additionally, reducing the sample volume delays pathogen detection by more than 2 h.

Solorzano-Santos et al. assessed the impact of blood sample volume on culture yield in paediatric patients, comparing microcultures (0.2 mL blood in 1.8 mL media) to standard cultures (2 mL blood in 18 mL media) and found no significant difference, although their study used non-automated systems and did not evaluate TTP [9]. In contrast, the present study, conducted with an automated blood culture system and fixed media volume, is, to our knowledge, the first from North India to address these questions in paediatric bacteraemia in vivo. At our institution, as in many paediatric units, the standard practice is to obtain at least 0.5 mL of blood for culture in aerobic bottles, as the recommendation for two sets of cultures to improve yield is largely based on adult data and lacks strong paediatric-specific evidence [17]. Parallel blood drawn from a peripheral site and a central line may help distinguish between true bacteraemia from contaminants [18]. However, the optimal blood volume for paediatric blood cultures remains unclear, with prior studies reporting inconsistent correlations between blood volume and bacterial isolation rates [19,20]. Although increasing the number of bottles may theoretically improve organism recovery independent of volume, as suggested by other studies, we observed that there was no significant difference in TTP and pathogen isolation rates between samples with <0.5 mL and those with >0.5 mL of blood [21]. 

In our prospective study, we analysed the TTP of 78 positive blood cultures, with most CoNS isolated within 36 h. The shortest TTP was seen in *Klebsiella pneumoniae *(4.18 h), and the longest in *Micrococcus* (39.25 h). Our data align with previous studies, suggesting that the most definitive pathogens, including *Staphylococcus aureus,* *Klebsiella pneumoniae*, and *Escherichia coli,* were identified within 36 h, confirming their role in severe sepsis [22,23].

Approximately 48% of positive blood cultures yielded CoNS, consistent with recent studies [24,25]. Positive CoNS cases in our study were more likely to be true pathogens, with all pathogenic isolates flagging positive within 36 h, while contaminants like *Micrococcus* flagged beyond it. Clinical findings supported CoNS as a true pathogen in patients with severe respiratory distress, low birth weight, and immunocompromised status. Additionally, we observed significant differences in TTP between BSIs caused by drug-resistant and drug-susceptible pathogens, especially in cases involving methicillin-resistant *Staphylococcus aureus* (MRSA), extended-spectrum beta-lactamase (ESBL)-positive Enterobacteriaceae, and extensively drug-resistant (XDR) *Acinetobacter baumannii,* with drug-resistant pathogens exhibiting earlier positivity. These findings support the use of TTP for early detection of drug-resistant isolates, enabling clinicians to adjust treatment earlier. Most BSI isolates were detected within 36 h, reinforcing the recommendation for antibiotic de-escalation after 48 h [26]. The clinical significance of TTP varied by species, with Gram-positive cocci, *Candida *spp.*,* and Gram-negative bacilli showing optimal TTP values of ≤18.17 h, ≤18.45 h, and ≤11.39 h, respectively. An optimal TTP cutoff of 18.17 h for CoNS isolates helps distinguish true infections from contaminants, in line with Lai et al.'s study [27].

Limitations 

This study has several limitations. Being a single-centre study, the findings may not be generalizable to other settings. The small number of suboptimally filled bottles limited the statistical power to detect significant differences in pathogen recovery. Additionally, possible variability in sample collection methods and operator technique could have influenced the volume of blood collected.

## Conclusions

This study emphasizes the importance of optimizing blood draw volumes for blood cultures in children, highlighting the balance between sufficient sample collection and minimizing discomfort for paediatric patients. Furthermore, the study underscores that Gram-negative organisms exhibited a shorter TTP compared to Gram-positive and fungal isolates, reinforcing the utility of TTP as a potential prognostic and diagnostic tool in BSIs. By implementing a quality improvement initiative focused on blood volume monitoring and clinician feedback, the study observed an increase in appropriately filled culture bottles. This highlights the critical need for routine monitoring to ensure accurate diagnoses and reduce both patient discomfort and economic losses from underfilled blood culture bottles.
